# Identifying novel Plasmodium falciparum erythrocyte invasion receptors using systematic extracellular protein interaction screens

**DOI:** 10.1111/cmi.12151

**Published:** 2013-05-13

**Authors:** S Josefin Bartholdson, Cécile Crosnier, Leyla Y Bustamante, Julian C Rayner, Gavin J Wright

**Affiliations:** 1 Cell Surface Signalling Laboratory, Wellcome Trust Sanger InstituteHinxton, Cambridge, CB10 1HH, UK; 2 Malaria Programme, Wellcome Trust Sanger InstituteHinxton, Cambridge, CB10 1SA, UK

## Abstract

The invasion of host erythrocytes by the parasite *Plasmodium falciparum* initiates the blood stage of infection responsible for the symptoms of malaria. Invasion involves extracellular protein interactions between host erythrocyte receptors and ligands on the merozoite, the invasive form of the parasite. Despite significant research effort, many merozoite surface ligands have no known erythrocyte binding partner, most likely due to the intractable biochemical nature of membrane-tethered receptor proteins and their interactions. The few receptor–ligand pairs that have been described have largely relied on sourcing erythrocytes from patients with rare blood groups, a serendipitous approach that is unsatisfactory for systematically identifying novel receptors. We have recently developed a scalable assay called AVEXIS (for AVidity-based EXtracellular Interaction Screen), designed to circumvent the technical difficulties associated with the identification of extracellular protein interactions, and applied it to identify erythrocyte receptors for orphan *P. falciparum* merozoite ligands. Using this approach, we have recently identified Basigin (CD147) and Semaphorin-7A (CD108) as receptors for RH5 and MTRAP respectively. In this essay, we review techniques used to identify *Plasmodium* receptors and discuss how they could beapplied in the future to identify novel receptors both for *Plasmodium* parasites but also other pathogens.

## Introduction

Malaria is a devastating infectious disease caused by parasites of the *Plasmodium* genus, of which *Plasmodium falciparum* is responsible for approximately one million deaths annually (Murray *et al*., 2012). All of the symptoms and pathology of the disease are caused by the asexual blood stage of the infection which is initiated when a merozoite invades a host erythrocyte and multiplies before bursting the infected red blood cell to release up to 32 more invasive merozoites (Cowman and Crabb, 2006). While invasion in itself is a fascinating cellular biological event, it is also a conceptually attractive target for intervention, particularly a vaccine. Indeed, merozoites are – albeit briefly – directly exposed to the host humoral immune system and passive transfer of purified immunoglobulins from immune to non-immune individuals has been shown to significantly decrease parasitaemia (Cohen *et al*., 1961).

Merozoites are pear-shaped cells containing in their apical protuberance specialized secretory organelles, which release their contents of membrane-tethered ligands in an ordered schedule during the invasion process (Singh *et al*., 2010; Gaur and Chitnis, 2011). Erythrocyte invasion has been separated into discrete stages based on video microscopy and electron micrograph images (Dvorak *et al*., 1975; Aikawa *et al*., 1978; Gilson and Crabb, 2009). The initial contact between the merozoite and erythrocyte is believed to be reversible and can occur anywhere on the merozoite surface. The merozoite then orientates itself, bringing its apical end into contact with the erythrocyte membrane to form an electron-dense junction at the point of contact between the two membranes. The junction opens out into a ring-like structure which is propelled towards the basal end of the merozoite by the action of an actin–myosin motor within the parasite. The relative movement of the parasite and erythrocyte membranes results in the complete internalization of the merozoite which is enclosed by a parasitophorous vacuole, a membrane that is derived, in part, from the erythrocyte surface (Cowman *et al*., 2012; Harvey *et al*., 2012).

Invasion is clearly a complex cellular process and requires the co-ordinated interactions between many proteins both on the parasite and host surfaces (Gaur and Chitnis, 2011). Although the > 50 known proteins present at the merozoite surface could have many biological functions, including modulating the immune response, the majority of them have been suggested to bind directly to the erythrocyte surface (Cowman and Crabb, 2006; Cowman *et al*., 2012). Despite being an area of intense study, there are currently only six known interactions between *P. falciparum* merozoite protein ligands and human erythrocyte receptors. Five of these involve members from two main families of invasion ligands: the erythrocyte binding-like (EBL) and reticulocyte binding-like homologue (RH) proteins (Tham *et al*., 2012). Given the global medical importance of malaria and the interest in merozoite surface proteins as vaccine candidates, why have so few interactions been identified between host receptors and *P. falciparum* merozoite surface proteins? Here, we suggest that the answers are partly due to the technical challenges of working with membrane-embedded receptor proteins, the typically weak interaction strengths of extracellular protein : protein interactions and the difficulties of expressing *Plasmodium* proteins in a functionally active recombinant form. We will highlight experimental factors that should be considered and suggest solutions that may help in identifying novel membrane-tethered receptors not only for *Plasmodium* proteins but also other pathogens.

## The challenges of identifying novel extracellular protein interactions

Despite their central role in many biological processes and importance as both drug and vaccine targets, detecting protein interactions mediated by membrane-embedded receptor proteins remains technically challenging. Membrane proteins are often amphipathic, containing on the same molecule a highly hydrophilic glycan coat as well as a hydrophobic transmembrane-spanning segment, which makes them difficult to solubilize in detergents while retaining their native conformation. Their relatively low abundance (typically 10^4^ to 10^5^ copies per cell), and presence of structurally critical post-translational modifications such as disulfide bonds also make them biochemically difficult to manipulate or recapitulate in a recombinant form (Wright *et al*., 2010). Extracellular protein interactions – particularly those between membrane-embedded receptor proteins – typically have equilibrium dissociation constants in the micromolar range, equating to kinetic half-lives of just fractions of a second when measured monomerically. The transient nature of extracellular interactions seems to have evolved to enable interacting cells to retain their independent motility necessary for function (Wright, 2009). Popular protein interaction methods that can be employed in a scalable manner are generally unsuitable for the identification of interactions involving membrane-embedded receptor proteins. For example, the presence of a hydrophobic transmembrane region and disulfide bonds are not compatible with the yeast-2-hybrid method, which necessarily requires the interacting proteins to be expressed as soluble active proteins in the reducing environment of the yeast nucleus. Similarly, biochemical purification methods and analysis by mass spectrometry require long and stringent washing steps, which are not suitable for detecting highly transient interactions. Given these challenges, alternative approaches had to be used to identify erythrocyte receptors for *P. falciparum* merozoite surface proteins.

## How were the known *Plasmodium* receptors identified?

The first interaction to be discovered between a *P. falciparum* merozoite surface ligand and an erythrocyte receptor was that between EBA175 and the most abundant membrane-tethered protein on the erythrocyte cell surface, GLYCOPHORIN A (GYPA) (Orlandi *et al*., 1992). EBA175 had previously been identified as a major parasite ligand capable of binding human erythrocytes in a trypsin and neuraminidase-dependent manner, suggesting its receptor was a sialylated protein (Camus and Hadley, 1985). The inability of EBA175 to bind M^k^M^k^ or Tn erythrocytes which lack GYPA and GYPB, or terminal sialyl and galactosyl residues on O-linked oligosaccharide structures respectively, implicated GYPA or GYPB as likely receptors. These observations were supported by the ability to co-immunoprecitate EBA175 and GYPA, an experiment likely made possible because of the sheer abundance of GYPA on red blood cells and the large number of O-linked glycosylation sites on this protein that would substantially increase the avidity of the GYPA-EBA175 interaction (Orlandi *et al*., 1992). The specificity of this interaction was further confirmed by the observation that EBA175 was unable to bind En(a-) erythrocytes (which lack GYPA, but still have sialylated GYPB) (Sim *et al*., 1994).

Following on from this initial discovery, additional members of the EBA family, EBA140 and EBL1, were shown to bind other glycophorins. In the case of EBA140, binding to glycophorins, including GYPC, was shown using far-Western blots of erythrocyte ghost preparations with wild-type and *EBA140*-deficient parasite culture supernatants (Maier *et al*., 2003). The binding specificity of EBA140 for GYPC was established by using erythrocytes from blood donors that either lacked GYPC (Leach phenotype) (Lobo *et al*., 2003), or lacked specific exons in the *GYPC* gene (Yus and Gerbich phenotypes) (Lobo *et al*., 2003; Maier *et al*., 2003). Similarly, GYPB-deficient (S-s-U-) erythrocytes were used to demonstrate that GYPB is the receptor for EBL1 (Mayer *et al*., 2009).

In addition to the three EBA-glycophorin interactions, an erythrocyte receptor has also been identified for RH4, a member of the reticulocyte binding-like (RH) family of parasite ligands. RH4 binding to erythrocytes was shown to be neuraminidase resistant, but trypsin and chymotrypsin sensitive (Gaur *et al*., 2007; Tham *et al*., 2009), an enzyme profile that – along with many other receptors – matches complement receptor 1 (CR1). Further work showed that anti-CR1 antibodies or soluble recombinant CR1 were able to inhibit binding of RH4 to erythrocytes, and that the affinity of the interaction between RH4 and CR1 was relatively weak with an equilibrium dissociation constant (*K*_D_) of 2.9 μM (Tham *et al*., 2010).

Although successful, the past approaches used to identify interactions between merozoite ligands and erythrocyte receptors have mostly relied on the existence and acquisition of erythrocytes carrying mutated blood group antigens and rational hypothesis-driven candidate receptor selection based on the sensitivity of parasite proteins binding to enzyme-treated erythrocytes. These approaches rely on the fact that the human erythrocyte surface is relatively simple and both biochemically and genetically well characterized. Comprehensive proteomic cataloguing (Pasini *et al*., 2006), monoclonal antibody typing (Mason, 2001) and decades of investigating blood transfusion incompatibilities have led to a vast resource of information about the biochemical composition and identification of even extremely rare genetic variants within the human population.

Despite these resources, however, obtaining erythrocytes that are specifically deficient in one particular receptor is not straightforward. The extreme rarity of many blood groups, the limited shelf life of cellular biopsies and the possibility that the absence of some receptors might be incompatible with normal erythrocyte function, make this approach generally unsuitable for systematically identifying new ligand-receptor pairs. Similarly, using binding sensitivity to proteases such as trypsin and chymotrypsin, which have broad substrate specificities, is only rarely helpful because a large number of receptors are sensitive to these enzymes. The identification of new interactions has also been hampered by the technical difficulties in expressing *Plasmodium* proteins in a biochemically active recombinant form that can subsequently be used as binding probes (Birkholtz *et al*., 2008). All these specific limitations, coupled with the general technical difficulty in identifying extracellular protein interactions described above suggest that the application of new methods and approaches may be helpful to identify novel erythrocyte receptors for *Plasmodium* merozoite surface proteins for which interaction partners are not yet known.

## AVEXIS: a scalable and systematic assay to identify novel extracellular protein interactions

**Fig 1:**
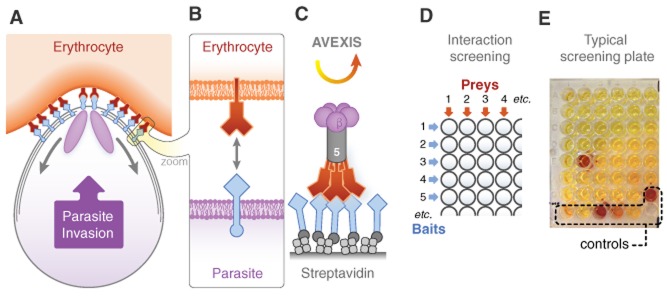
Identifying novel *Plasmodium* receptors by AVEXIS.A. Schematic representation of erythrocyte invasion by the malarial parasite highlighting the extracellular protein interactions between parasite ligands and erythrocyte receptors.B. Cartoon of interacting cell surface receptors between an erythrocyte receptor (red) and a parasite ligand (blue).C. The AVEXIS assay detects direct interactions between soluble recombinant proteins representing the entire ectodomain of cell surface receptors. Here, the erythrocyte receptors are expressed as the pentamerized β-lactamase-tagged ‘preys’ and the parasite ligands as monomeric biotin-tagged ‘baits’. The baits are captured on a streptavidin-coated solid phase, such as a microtitre plate or glass slide and probed with the preys. A brief wash is performed and captured preys are detected by addition of a β-lactamase substrate, nitrocefin which hydrolyses a yellow substrate into a red product, indicating a positive interaction.D. AVEXIS involves the systematic screening within libraries of both baits and preys in an all-versus-all matrix of direct binding tests.E. An image of a typical screening plate. Each well contains a different bait protein screened with a single prey. A positive interaction was observed in well E2 and control interactions are indicated within the dotted line.

To address the problems associated with identifying novel low affinity extracellular protein interactions, an assay named AVEXIS (for AVidity-based EXtracellular Interaction Screen) was developed (Bushell *et al*., 2008). AVEXIS detects highly transient direct interactions between recombinant proteins consisting of the entire ectodomain regions of secreted or cell surface receptor proteins expressed as soluble fragments in mammalian cells. Each protein is expressed as a monomeric biotinylated bait and a highly avid enzyme-tagged pentameric ‘prey’. The biotin tags are enzymatically added to a specific lysine residue using the BirA enzyme so that the baits can be captured in an orientated fashion on streptavidin-coated solid phases such as a microtitre plate (Bushell *et al*., 2008) or glass slide (Sun *et al*., 2012); these bait arrays are then systematically probed for interactions using the preys. The pentamerization of the prey proteins is achieved using a short peptide from the cartilage oligomeric matrix protein (COMP) that increases the local concentration of the ectodomain region, mimicking the clustered receptor proteins as would be found within the plasma membrane. This additionally increases the overall binding avidity so that interactions which would (monomerically) have half-lives of just a few seconds are now increased to ten of minutes to several hours (Bushell *et al*., 2008) permitting their detection (Fig. 1).

This assay requires the construction of a library of proteins within which interaction screens are performed to detect interactions. Because interactions will only be detected between proteins that are correctly folded, the entire extracellular domain of each protein is expressed to preserve binding sites and a mammalian expression system is used to ensure the addition of appropriate post-translational modifications such as the structurally critical disulfide bonds and glycans. The assay is highly generic and is suitable for any class of secreted proteins or membrane receptors that can be expressed as soluble ectodomains. It has been used to identify cell surface receptor interactions that are important for early vertebrate development (Martin *et al*., 2010), neural recognition processes (Sollner and Wright, 2009) and for the recognition of myoblasts that will subsequently fuse to form multinuclear skeletal muscle fibres (Powell and Wright, 2011).

## Applying AVEXIS to the problem of merozoite – erythrocyte recognition

The AVEXIS approach has recently been applied to systematically screen for receptor–ligand pairs involved in the recognition of the host erythrocyte by the blood stage of the malaria parasite (Crosnier *et al*., 2011; Bartholdson *et al*., 2012). For this purpose, two protein libraries representing the cell surface receptor repertoires of the human erythrocyte and the *P. falciparum* merozoite were compiled. The erythrocyte receptor library consisted of forty cell surface proteins that were detected in an in-depth proteomic catalogue of human erythrocyte ghost preparations (Pasini *et al*., 2006) and for which the extracellular region could be expressed as a soluble fragment (Crosnier *et al*., 2011). To address the technical challenges of expressing *Plasmodium* proteins recombinantly, codon-optimized expression constructs containing an exogenous signal peptide and in which N-linked glycosylation sequons were mutated to prevent inappropriate glycosylation were expressed in mammalian cells (Crosnier *et al*., 2011; Bartholdson *et al*., 2012). Of the 51 *P. falciparum* merozoite surface and secreted proteins that were originally selected from the literature and proteomic studies (Sanders *et al*., 2005; Cowman and Crabb, 2006; Gilson *et al*., 2006), over 75% of them could be expressed at usable levels (C.C., J.C.R. and G.J.W., manuscript submitted). These two protein libraries were systematically screened by AVEXIS to successfully identify two novel receptor ligand pairs: Basigin-PfRH5 and Semaphorin-7A-PfMTRAP.

## Basigin, an RH5 receptor that is essential for the invasion of *P. falciparum*

RH5 differs from the other members of the RH and EBA families in that it is much shorter and is predicted to be secreted, rather than tethered within the merozoite plasma membrane. Perhaps most importantly and unlike the other EBAs and RHs, *RH5*-deficient parasites cannot be isolated, suggesting an essential role in blood stage growth (Hayton *et al*., 2008; Baum *et al*., 2009). RH5 is expressed by all tested strains of *P. falciparum* and is localized to the merozoite rhoptries (Rodriguez *et al*., 2008; Baum *et al*., 2009). However, during invasion, it is observed at the moving junction where it colocalizes with RON4 and AMA1 (Baum *et al*., 2009). Genetic crosses have identified *RH5* as a gene responsible for the species tropism of invasion (Hayton *et al*., 2008) and its expression was positively correlated with parasitaemia in Gambian clinical isolates (Gomez-Escobar *et al*., 2010). RH5 is known to interact with another essential protein called Ripr, which is localized to the micronemes (Chen *et al*., 2011). Taken together, these studies suggested that RH5 could play an important role in erythrocyte invasion and the identification of its specific receptor might help to understand its role.

Using AVEXIS, the receptor for RH5 was identified as basigin (CD147), an erythrocyte cell surface protein that carries the Ok blood group antigen; as expected, RH5 and basigin directly interacted with low affinity (*K*_D_ of 1.1 μM). Perturbing the interaction between RH5 and basigin either biochemically or genetically potently inhibited invasion. Importantly, these inhibitory effects were observed across all tested strains demonstrating that the RH5-basigin interaction was both essential and universally required (Crosnier *et al*., 2011). In addition, antibodies to RH5 elicited by either viruses (Douglas *et al*., 2011) or a recombinant RH5 protein (Bustamante *et al*., 2013) could block invasion in all tested strains. Together, these data advance the candidature of RH5 as a blood-stage vaccine candidate.

Basigin expression is not restricted to erythrocytes, and the protein owes its name ‘**bas**ic **i**mmuno**g**lobul**in** superfamily’ to its widespread expression on cells of haematopoetic, epithelial, and endothelial origin (Miyauchi *et al*., 1990). Basigin has a range of documented functional roles and endogenous interaction partners (Muramatsu and Miyauchi, 2003; Iacono *et al*., 2007; Kennedy and Dewhirst, 2010), but its functional role on erythrocytes is not clear. It might have an ‘anti-adhesive’ role in splenic erythrocyte release since the administration of F(ab′)_2_ fragments of an anti-basigin monoclonal antibody in mice resulted in selective trapping of red blood cells within the splenic red pulp (Coste *et al*., 2001). A key structural feature of basigin is its highly conserved transmembrane-spanning region, which – unusually – contains a charged glutamic acid residue. This charged amino acid was found to be required for the correct targeting of the multispan monocarboxylate transporters MCT1 and MCT4 to the plasma membrane (Kirk *et al*., 2000). MCT1 is expressed on the surface of human erythrocytes (Pasini *et al*., 2006) where it functions primarily as an l-lactate-protein cotransporter. The role of basigin on erythrocytes is therefore likely to be a chaperone to ensure correct membrane localization and regulate the activity of MCT1.

## Semaphorin-7A: an erythrocyte receptor for *P. falciparum* MTRAP

Using a similar screening approach, Semaphorin-7A was identified as a receptor for the merozoite-specific thrombospondin-related anonymous protein, MTRAP. The TRAP-like family protein is believed to provide a crucial link between the host cell and the parasite’s cytoplasmic actin–myosin motor that powers the invasion of the parasite but no host binding partners had been identified so far. Similar to RH5, MTRAP is believed to be refractory to genetic deletion, suggesting a critical role in erythrocyte invasion (Baum *et al*., 2009). The interaction between MTRAP and Semaphorin-7A was also shown to be weak, with a *K*_D_ of 1.18 μM and this interaction did not involve the presence of glycans on the receptor (Bartholdson *et al*., 2012). The function of the MTRAP-Semaphorin-7A interaction in parasite invasion is also unclear since the addition of antibodies to either the ligand or receptor did not cause any observable perturbations in *in vitro* parasite invasion assays (Bartholdson *et al*., 2012) in line with other studies (Baum *et al*., 2009; Uchime *et al*., 2012). Consistent with this, none of the eight known naturally occurring human variants of Semaphrorin-7A affected the binding of MTRAP and it is therefore unlikely that these polymorphisms have played any protective role against *Plasmodium* infections in human populations (Bartholdson *et al*., 2012).

Semaphorin-7A (CDw108) is a GPI-linked protein carrying the John-Milton-Hagen blood group antigen (Mudad *et al*., 1995) whose expression has also been observed on activated leucocytes as well as in the spleen, thymus, testis, placenta, brain and spinal cord (Xu *et al*., 1998; Yamada *et al*., 1999). Semaphorin-7A has an important role in the development of both the nervous and immune systems where it promotes axon outgrowth through integrins (Pasterkamp *et al*., 2003), and has also been implicated in the regulation of T-cell function (Czopik *et al*., 2006; Suzuki *et al*., 2007; Gras *et al*., 2013), bone cell differentiation (Delorme *et al*., 2005), spreading and dendricity in human skin melanocytes (Scott *et al*., 2008), nerve regeneration and inflammation processes in the cornea (Namavari *et al*., 2012), and GnRH-1 neuron development (Messina *et al*., 2011). The functional role of Semaphorin-7A on erythrocytes remains unclear. Interestingly, some individuals acquire the JMH-negative phenotype as they become older due to the gradual loss of Semaphorin-7A expression from the surface of their erythrocytes (Seltsam *et al*., 2007). This reduction in erythrocyte cell surface Semaphorin-7A expression is not thought to be transcriptional since erythrocytic mRNA levels appear unaffected suggesting a post-translational or active shedding mechanism (Seltsam *et al*., 2007).

## Conclusions

**Fig 2:**
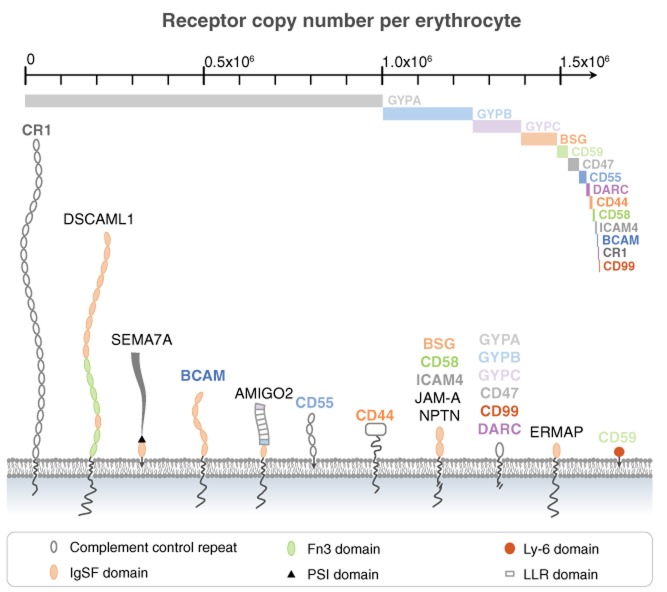
The cell surface receptor repertoire of the human erythrocyte. A schematic structural representation of the human erythrocyte cell surface receptor repertoire drawn approximately to scale together with the receptor abundances, where known, shown as bars on the scale. The figure contrasts large receptors such as CR1, which project 85 nm away from the membrane, but, at 1000 copies per cell, are relatively rare whereas the much smaller glycophorins are vastly more abundant, containing up to a million copies per cell in the case of GYPA.

The application of AVEXIS to the problem of erythrocyte-merozoite recognition has led to the recent identification of two novel erythrocyte receptors for *P. falciparum* parasites, the first receptors to be identified by an unbiased screening approach. It is notable that unlike Glycophorins A, B and C, neither of these new receptors are erythrocyte-restricted. This means that the RH5-Basigin and MTRAP–Semphorin-7A interactions cannot be used for erythrocyte recognition, but rather play adhesive or mechanical roles during invasion. This in turn implies that *P. falciparum* merozoites use different receptor interactions for different purposes – not all interactions are functionally equivalent and interchangeable, as is sometimes assumed. This point is reinforced by comparing the size and abundance of the known invasion receptors, which range in size from 85 nm (CR1; Weisman *et al*., 1990) to just a few nm (∼ 70 amino acids for extracellular GYPA), and in copy number from approximately one million (GYPA) to just a few thousand (CR1) (Anstee, 1990) (Fig. 2). This makes for extreme differences in the accessibility of each receptor to the merozoite, and it is reasonable to imply that different receptors may therefore have different functions or act at different stages during invasion. The differences in abundance of known receptors also emphasize the challenges that lie ahead. The identification of GYPA as a receptor was clearly facilitated by its abundance on the erythrocyte, but this will not hold true for interactions that await discovery. Further applications of AVEXIS may identify new interactions, but completely new approaches will be needed for other classes of erythrocyte protein that are not suitable for this technique such as those that are glycan dependent or multipass transmembrane proteins. Indeed, multipass proteins are a significant feature of the erythrocyte surface with over 50 different proteins being identified in mass spectrometry-based surveys of human erythrocytes (Pasini *et al*., 2006) with some, such as the Band 3 anion transport protein, being highly abundant, with copy numbers similar to that of GYPA at approximately one million per cell (Anstee, 1990). Other new technologies such as the rapidly improving methods in mass spectrometry applied to cell surface proteins (Wollscheid *et al*., 2009) and stem cell-based genetic manipulation and differentiation protocols (Bei *et al*., 2010) will be helpful to identify these interactions. Together, these technologies will lead to a fuller mechanistic understanding of invasion which will be important in the rational development of therapeutics or vaccines designed to prevent parasite entry, eventually reducing the humanitarian burden of malaria.
